# Surgical simulation in emergency management and communication improves performance, confidence, and patient safety in medical students

**DOI:** 10.1080/10872981.2025.2486976

**Published:** 2025-04-08

**Authors:** Mazlum Baris, Nils von Schaper, Hannah Sofie Weis, Klaus Fröhlich, Christian Rustenbach, Anne Herrmann-Werner, Christian Schlensak, Christoph Salewski

**Affiliations:** a Department of Thoracic and Cardiovascular Surgery, University Medical Center Tübingen, Tübingen, Germany; b Medical Faculty, University of Tübingen, Tübingen, Germany; c TIME Tübingen Institute for Medical Education, Tübingen, Germany

**Keywords:** Emergency communication, bleeding control, emergency management, intraoperative emergency, phantom trainer, simulation, medical education, surgical education

## Abstract

**Introduction:**

This study aims to enhance the confidence and operational safety of 5th-year medical students in the operating room (OR), addressing their corona pandemic gap in surgical training.

**Methods:**

We augmented the surgical curriculum focusing on pre-, intra-, and post-operative skills, centered around a phantom operation as a pre-test-retest simulation. We measured confidence to assist in surgery on a 5-level Likert-scale and monitored surgical performance metrics (skin-to-skin time, blood loss, blood and volume transfusion, complications, fatal outcome). Half the cohort was explicitly video trained in hemostasis, while the other half in emergency communication. Factual knowledge gains were assessed with online questionnaires. The groups served as reciprocal controls, as confidence (communication group) and surgical performance (bleeding group) were compared.

**Results:**

Initially, the pre-test performance of the 126 participants on the phantom operation was suboptimal, ranging from poor to mediocre. Notably, the retest outcomes demonstrated significant surgical performance improvements following the targeted lessons (e.g. blood loss pre-test 906 ± 468 mL, retest 292 ± 173 mL, *p* < 0.01, *n* = 35 teams), with the most pronounced enhancements observed in confidence and emergency communication skills (confidence pre-test 2.42 ± 0.52, retest 3.55 ± 0.64, *p* < 0.01, *n* = 35 teams). There is a strong tendency (*p* = 0.08) that the communication group (1.28 ± 0.53) had higher gains in confidence than the bleeding group (0.997 ± 0.4) with a moderate effect size (Cohen’s D = 0.6). Students reported increased confidence in assisting in surgery compared to their initial self-assessments. These results show that the structured exposure to a pre-test-retest phantom operation substantially elevates students’ capability to act safely and assertively in the OR.

**Discussion:**

This approach appears to foster a justified increase in confidence and surgical performance, potentially elevating patient safety among students and residents in training.

## Introduction

The operating room (OR) is a difficult environment for medical students in which they are initially ‘unconsciously incompetent’, as J Flower has put it [1]. Conventional approaches to medical education have proven inadequate, especially during the corona pandemic [2]. Medical education was taken from the bedside to the screen, which is limiting in internal medicine, but was deleterious in surgical education as practical hands-on training was suspended and trainees were reassigned to coronavirus patient-care regions [3,4]. Then, confronted with surgical tasks, the realization of being consciously incompetent in the OR can be discouraging for young colleagues. We believe that traditional lectures are unable to teach the manual skills necessary to make students consciously competent. However, becoming unconsciously competent (like riding a bike) is only feasible with many repetitions [5,6]. Therefore, we aim at becoming ‘consciously competent’ within this study. Within our conceptual framework, we believe that confidence to assist in surgery and surgical performance metrics are interdependent variables. We postulate that surgical confidence goes with increased manual skill, the ability to mix and understand orders, demonstrate active followership, but be able to communicate, and lead a team when necessary. Of course, an overly motivated and unjustified confident student might perform poorly in surgery, as a meek and careful one might excel in performance metrics without building justified confidence. We anticipate that improved surgical performance metrics back up students’ confidence to be willing to assist in surgery. Vice versa we predict that confident students will do better in surgical performance metrics. In this study, there are two reciprocal study groups: We hypothesize that participants trained in emergency communication will report higher confidence levels, and that students trained in hemostasis will be better in surgical performance metrics. Thus, we can measure both confidence and competence and assess interdependency. Literature research revealed that manual skills can be effectively taught and retained [7,8]. The discrepancy between students’ theoretical knowledge and their practical skills, especially in emergency situations in the OR, requires an innovative approach to training [9]. Other educators, as well as we, have set confidence in surgical emergencies as an educational objective [10–13]. As the national competency-based learning objectives catalog [14] calls for the teaching of competencies, we propose a novel curricular initiative to address this gap. Adherence to standards such as the WHO surgical checklist is useful in preparing for critical situations [15], and simulation is an ethical imperative [16].

## Materials and methods

### Materials

#### Phantom trainer design and functions

**Figure 1.**
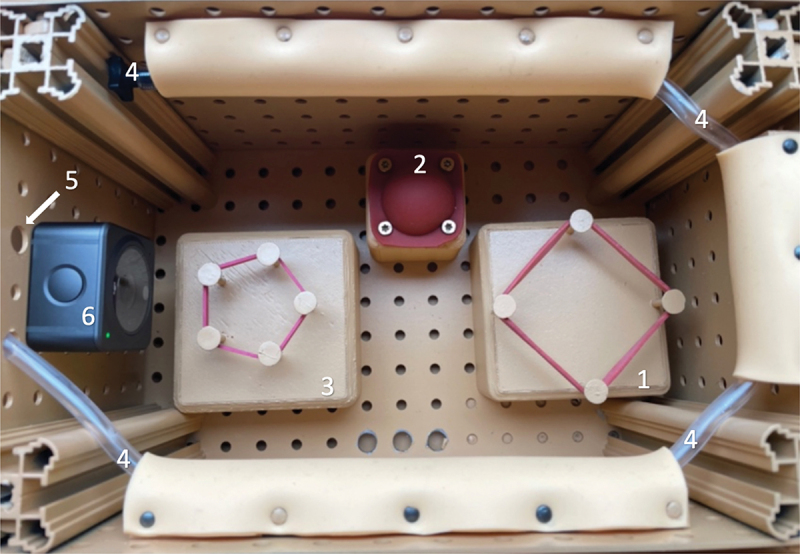
Surgical phantom trainer: 1. Knotting task, 2. Preparation task, 3. Sewing task, 4. Hose for diffuse bleeding, 5. Vent for Hose for surgical bleeding, 6. Camera.

Our phantom trainer mimics an arbitrary body cavity (Figure 1). It incorporated three sequential tasks: knot-tying, dissecting, and suturing. Three liquid-filled balloons were placed adjacent to the tasks to constrain the range of motion. Damaging these balloons was seen as a complication.

#### Simulated bleeding mechanisms

##### Surgical bleeding

Emanating from a tube at a rate of approximately 100 ± 10 mL/min, this ‘surgical’ bleeding required immediate intervention by the surgical team (Figure 1, no. 5). This required to momentarily abandon the surgical task, employing clamps or sutures effectively. Anesthesiological measures were supportive but not effective to stop the surgical bleeding.

##### Diffuse bleeding

50 ± 5 mL/min oozed from a tube embedded in a sponge, indicative of coagulation issues. Unlike the surgical bleeding, it could only be managed by the anesthetist through i.v. clotting factors (Figure 1, no. 4). This bleeding was not amenable to surgical intervention, necessitating clear communication between the surgical team and the anesthetist.

The anesthetist was provided with a 1-liter flask with red dye, symbolizing the patient’s blood volume. A pump was connected to the phantom trainer. Activation of the pump simulated the dynamic challenge of managing blood loss, with the depletion of the flask.

Three lines went into the flask, allowing for the administration of volume, blood, and clotting factors. The level of the flask indicated both the patient’s blood pressure and hemoglobin levels.

#### Provision of surgical material

Each group was equipped with a full array of surgical and anesthesiological material for an immersive experience. A detailed inventory is listed in table S2 of the appendix.

### Methods

#### Setting

**Figure 2.**
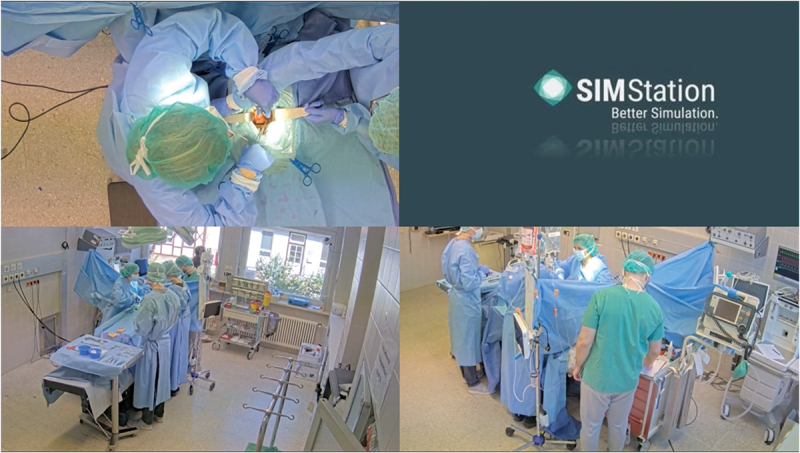
Phantom operating room setting. Multi angle supervision allowed insight and feedback.

The simulation took place at the Tübingen PAtient Safety Simulator (TüPASS) and the Tübingen Institute for Medical Education (TIME), which house state-of-the-art simulation theaters (Figure 2).

#### Study design

**Figure 3.**
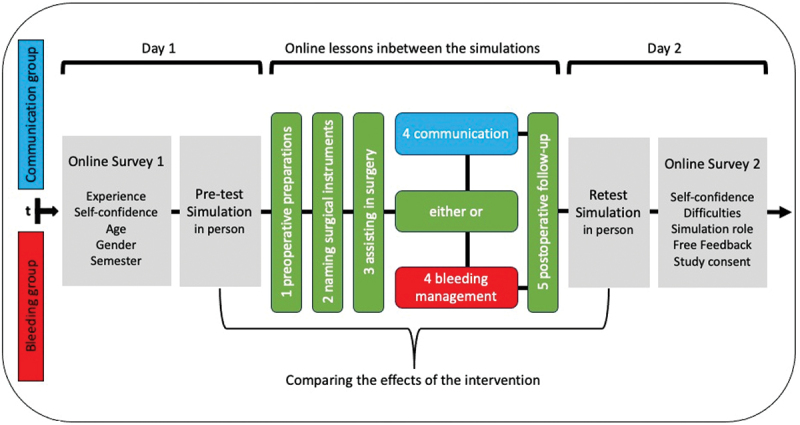
Study course. Students were assigned to a communication group or bleeding group. Their study course was identical, despite a special training in emergency communication or bleeding control, respectively.

We conducted this study with 5th-year medical school students during the summer semester 2023 at the Department of Thoracic and Cardiovascular Surgery, Medical Faculty of our University Hospital. A reciprocal control trial design explored the impact of a surgical phantom operation on their subjective confidence and surgical performance (Figure 3). We could not assign students to groups through propensity score matching, as the groups of 4–6 students and their date of attendance were set by the faculty and not by the authors. Therefore, we adopted a quasi-randomization approach.

The course’s focus switched from ‘bleeding control’ to ‘emergency communication’ after the midpoint of the semester distinguishing the two study groups. The simulation maintained a level of fidelity that ensured all tasks could be approached without prior specialized knowledge.

##### Assessment and feedback

Students completed online surveys before the first of two phantom operations and after the second. These questionnaires aimed to assess demographic data, surgical experience, changes in the students’ subjective confidence, and perceptions of their surgical performance (Figure 3, first grey box). With a five-point Likert scale (ranging from 1 ‘poor’ to 5 ‘excellent’), we asked ‘With your current skills, how confident do you feel about being the first assistant in surgery?’ In each week we allowed three student teams. The bleeding group was additionally taught in bleeding management but not in communication skills, whilst the communication group was taught vice versa.

#### Assignment of roles

Students were randomly assigned to be the surgeon, first assistant, surgical technical assistant, or anesthetist and grouped accordingly.

#### Data collection

Skin-to-skin time, blood loss, blood and clotting factors transfused, volume administered, complications, and fatal outcome were recorded.

#### Online lessons

For homework, the students were tasked with completing these online lessons prior to the retest:

1. Preoperative preparations (WHO Checklist)

2. Naming and use of surgical instruments

3. Assisting in surgery

4. *either* Bleeding management (bleeding group)

***or*** Emergency communication (communication group)

5. Postoperative follow-up (pain, wounds, thrombosis prophylaxis)

Each lesson began with a questionnaire, followed by an instructional video (all videos linked in the appendix), and concluded with a repeat-questionnaire. Achievable points are listed in the appendix.

#### Retest

Maintaining their initially assigned roles, students repeated the simulation within the same week. The session ended with a debriefing and a second completion of the online confidence questionnaire.

### Endpoints

**Figure 4.**
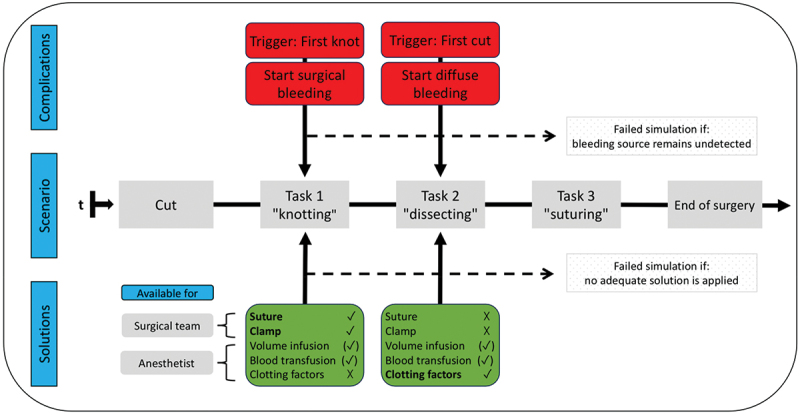
Sequence of the simulation: tasks and bleedings. “cut“and “end of surgery“ were carried out on the surgeon’s command. The first successful knot during task 1 was the trigger for the surgical bleeding. The first cut during task 2 was the trigger for the diffuse bleeding. Every possible supportive measure (green boxes) was available at any time. Effectiveness is labeled as: adequate √, supportive but not adequate (√), unsuccessful x. Simulation was failed if the bleeding source remained undetected and/or no solution was applied.

The simulation followed the scheme in Figure 4. It was deemed complete when the surgical tasks were successfully executed, and all bleedings were controlled. Alternatively, the operation concluded if the phantom patient succumbed to bleeding, or the surgical bleeding remained undetected. There was no time restriction.

#### Twofold educational goals

Surgical Management: To encourage the surgical team to find manual solutions to control surgical bleeding while simultaneously managing another task.

Team Communication: To ensure the team recognized and effectively communicated the onset of the diffuse bleeding, which required a non-surgical response.

As groups involved 3–5 participants, the sum of confidence scores within each group was divided by the number of participants.

### Request for long term results

One year after the program, we sent a further online questionnaire to the participants before they finish medical school. At this point we asked for their confidence to assist in surgery and perceived skill retention.

### Statistics

All available cases were analyzed with SPSS 28 (IBM Corp., Armonk, NY, USA). Continuous variables were visually tested for normal distribution (kurtosis and skew). Normally distributed data are reported as means and standard deviations (SD); Categorical data are reported as counts and percentages. For inferential statistical comparisons of categorical variables, the χ^2^ test was used. t-tests analyzed pre-test-retest comparisons. The distribution of the individual differences between pre- and retest scores in the bleeding and communication groups fulfilled criteria of normality for the overall test results. Data reporting complied with the EACTS statistical and data reporting guidelines [17].

#### Ethics committee’s approval

This study was reviewed and approved by the local ethics committee under the No. **841/2022BO2**. All participants gave written informed consent.

#### Reporting of results

The results are divided into key comparisons (A-E). Irrelevant differences were not mentioned. All numerical results are listed in table S4 of the appendix.

(A) Cohort Analysis: Overall Pre-test vs. Post-test

(B, C) Subgroup Performance: Pre-test vs. Post-test (B = bleeding, C = communication)

(D) Comparative Analysis: Change Scores between Subgroups (B and C)

(E) Post-intervention Retest: Subgroup Comparisons (B and C)

## Results

150 participants were invited in this study. 138 participated in the study. A total of 126 (100%) students submitted complete datasets (Table 1). The bleeding and communication groups served as reciprocal controls. Ex post structural equality was proven.

**Table 1.** Baseline parameters.

Quality	Bleeding group	Communication Group	P value
Number of participants	62	64	
Number of teams	19	16	
Age years	26.10 ± 3.2 years	26.09 ± 2.8 years	0.996*
Gender (women)	44 (71%)	39 (61%)	0.284^+^
Professional training	7 (11%)	8 (13%)	0.835^+^
Surgical electives	32 (52%)	37 (58%)	0.491^+^
Academic classes	10 (16%)	5 (8%)	0.153^+^
Scrub nurse fundamentals	19 (31%)	14 (22%)	0.194^+^
Other	6 (10%)	19 (30%)	0.003^+^
No experience	18 (29%)	7 (11%)	0.011^+^
Confidence (pre-test)	Poor (30) fair (42) moderate (29) good (18) excellent (4)		
	21 18 14 6 3	9 24 15 15 1	0.057*

*Independent samples t-test; ^+^ χ^2^ test.

### Confidence scores

**Figure 5.**
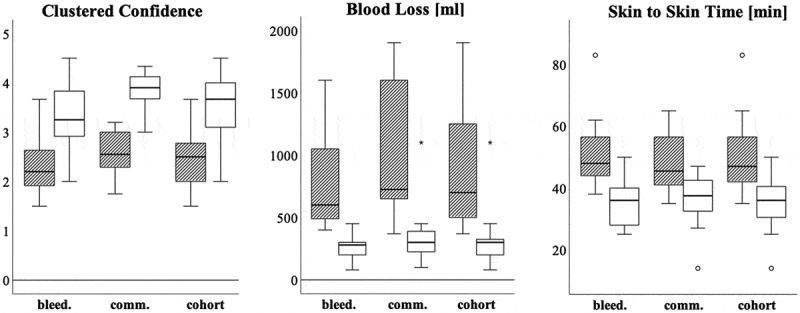
Boxplots of clustered confidence (left), blood loss (middle), and skin to skin time (right). Legend: striped = pretest, white = retest results.

- Overall Increase: The entire cohort showed significant improvement in confidence, with pre-test scores of 2.42 ± 0.52 increasing to 3.55 ± 0.64 in the retest (*p* < 0.01, Cohen’s D = 2.3, *n* = 35) (Figure 5, left).

(B)(C) Subgroup Gains: The bleeding subgroup’s confidence rose from 2.3 ± 0.6 points to an average of 3.29 ± 0.7 (*p* < 0.001, Cohen’s D = 2.461, *n* = 19), while the communication group saw a more substantial increase from 2.57 ± 0.41, reaching an average of 3.86 ± 0.38 (*p* < 0.001, Cohen’s D = 2.3, *n* = 16).

(D) Comparative Confidence: Group-wise comparison indicated a strong tendency (*p* = 0.08) for higher confidence gains in the communication-trained teams (1.28 ± 0.53) over the bleeding-focused teams (0.997 ± 0.4), with a moderate effect size (Cohen’s D = 0.6).

(E) The communication group notably achieved significantly higher retest confidence scores (3.86 ± 0.38) compared to the bleeding group (3.29 ± 0.7; *p* < 0.01, Cohen’s D = 0.61).

Most students, i.e., 71.4%, reported an increase in confidence, 20.6% reported no change, and 7.9% felt a decrease in confidence (Appendix figure S6).

### Surgical outcome scores

#### Blood loss, volume management, and transfusion

- The cohort substantially improved in reducing blood loss from an average of 906 ± 468 mL in the pre-test to 292 ± 173 mL in the retest (*p* < 0.01, Cohen’s D = 1.1; *n* = 35) (Figure 5, middle). They also had to substitute less volume (pre-test: 254 ± 152 mL; posttest: 163 ± 104 mL; *p* < 0.01, Cohen’s D = 1.07 *n* = 35), and the amount of blood transfusion was less in the posttest, as well (pre-test: 239 ± 202 mL; 42 ± 98 mL in posttest; *p* < 0.01, Cohen’s D = 0.83; *n* = 35).
- The bleeding subgroup had 533 mL less blood loss than in the pre-test (from 792 ± 376 mL down to 259 ± 103 mL, *p* < 0.001, Cohen’s D = 1.28), the amount of volume given decreased from 355 ± 142 mL to 126 ± 77 mL (*p* < 0.001, Cohen’s D = 1.43), and the amount of blood transfused fell from 192 ± 194 mL to 18 ± 29 mL (*p* < 0.001, Cohen’s D = 0.9).
- The communication subgroup had 710 mL less blood loss than in the pre-test (from 1,042 ± 539 mL down to 331 ± 228 mL; *p* < 0.001, Cohen’s D = 1.13), the amount of volume given decreased from 353 ± 166 mL to 206 ± 118 mL (*p* < 0.008, Cohen’s D = 0.76), and the amount of blood transfused fell from 294 ± 203 mL to 70 ± 138 mL (*p* < 0.006, Cohen’s D = 0.791).

#### Skin-to-skin time and fatal outcomes

- The entire cohort showed a notable reduction in skin-to-skin time, decreasing from an average of 49 ± 10 to 35 ± 8 min (*p* < 0.01, Cohen’s D = 1.3; *n* = 35) (Figure 5, right).
- There were no fatal outcomes in the retest, contrasting with nine in the pre-test (χ^2^ = 8.26 and *p* = 0.004; *n* = 35).

#### Recognition of the surgical bleeding

(A) Both surgical and diffuse bleedings were recognized and managed more quickly in the retest, with surgical bleeding recognition time dropping from 45 ± 28 to 28 ± 21 sec (*p* < 0.001, Cohen’s D = 0.5; *n* = 34). Diffuse bleeding recognition (180 ± 132 to 60 ± 59 sec; *p* < 0.001, Cohen’s D = 0.77; *n* = 28) also saw significant improvements.

#### Time to stop the surgical bleeding

- In the pre-test the cohort took 4.7 ± 3.8 min to stop the surgical bleeding, whereas in the retest they only took 1.4 ± 1.0 min (*p* < 0.001, Cohen’s D = 0.79; *n* = 33). One group did not recognize the surgical bleeding in the pre-test.
- The bleeding subgroup stopped the surgical bleeding 2.5 min faster in the retest (from 3.9 ± 2.9 to 1.3 ± 0.5 min; *p* < 0.001, Cohen’s D = 0.89; *n* = 19).
- The communication subgroup stopped the surgical bleeding 4 min faster in the retest (from 5.8 ± 4.7 to 1.7 ± 1.6 min; *p* < 0.001, Cohen’s D = 0.79; *n* = 16).

#### Time to stop the diffuse bleeding

- In the pre-test the cohort took 10.3 ± 7.5 min to stop the diffuse bleeding, whereas in the retest they only took 3.5 ± 3.6 min (*p* < 0.01, Cohen’s D = 0.77; *n* = 28).
- The bleeding subgroup stopped the diffuse bleeding 8.8 min faster in the retest (from 11.7 ± 8.7 to 2.8 ± 2.1; *p* < 0.001, Cohen’s D = 0.99; *n* = 18). (C) The communication subgroup stopped the diffuse bleeding 2.8 min faster in the retest (from 7.6 ± 4.1 to 4.8 ± 5.2; *p* = 2.53, Cohen’s D = 0.39; *n* = 16). (D) There is a strong tendency the bleeding group (*n* = 18) was better prepared to stop the diffuse bleeding than the communication group (*n* = 10) (*p* = 0.077, Cohen’s D = 0.73).

#### Miscellaneous

There was less talk in the retest, and the communication was more direct and effective. The class was rated excellent 1.2 ± 0.5 (*n* = 64; caveat: 1 ‘excellent’ to 5 ‘poor’). Similar classes at our faculty were rated 1.8 ± 1.0 average (*n* = 1,721). As free text feedback the participants reported they could familiarize themselves with the OR environment and calm their anxiety. Even surgically uninterested participants with unease in the OR felt more comfortable and gained fundamental insights in the operative way of work. There were even demands for more and different scenarios and expansion of our curriculum to a full week. Some participants complained that not every team member (instrumenting nurse, anesthetist) got to train manual skills. Overall, the amount of effort for the curriculum’s development was appreciated.

Finally, we asked the participants about their perception of the class on a 5-point Likert scale:

##### Confidence

43.4% and 38.2% report to have a good/very good idea about the division of work and responsibility in the OR. Instrument knowledge was reported to be fair 43.4% to good 28.7%. 44.1% to 27.9% dare to assist in surgery much or very much. Emergency communication was reported as mastered 42.6% to mastered well 33.1%. 27.2% to 43.4% feel fairly save or save in intraoperative hemostasis. 24.3 to 40.4% feel fairly save or save with postoperative care measures.

##### Helpfulness

To gain the class experience and benefit in confidence and competence we asked, what was perceived as helpful. 61.8% found the phantom trainer very helpful. 34.6% to 30.9% found a technical video on surgical knotting helpful or very helpful. The actual visit to a life OR case was mainly perceived as fairly helpful with 27.2%. 41.2% and 40.4 found the educational videos helpful to very helpful. 22.1% to 56.6% found the repetition of the scenario helpful to very helpful.

##### Difficulty

22.8% to 25.7% found the surgical tasks a little to fairly difficult. 19.1% to 28.7% had a little or fair difficulty in using the instruments. Resolving the intra-operative complications was perceived as a little difficult 30.1% to fairly difficult 29.4%. Intraoperative emergency communication was deemed a little 40.4% to fairly 21.3% difficult. Recognizing the complication was rated as a little 41.2% to fairly 19.1% difficult

### Results of online tests

Significant improvements were observed in the pretest-posttest scores across all five online lessons (*p* < 0.01). The questions, suggested answers, and graphed results can be seen in table S3 and figure S7 of the appendix.

### Long term skill retention

One year after the intervention, we offered the whole cohort (*n* = 126) a questionnaire to report their perceived confidence and long-term skill retention. Of them, *n* = 30 students responded and gave informed permission to analyze their answers.

#### Confidence

One year later their confidence is still on a comparable level as just after our intervention. Notably, there was no confidence-drop in this sample, which can be seen in Figure S6, left.

#### Surgical performance metrics

First, we could not repeat the program a whole year after the study has taken place with the same participants. Therefore, long-term surgical performance metrics (skin to skin time, blood loss etc.) cannot be reported. Instead, we asked for their self-reported skill levels which can be seen in Figure S6, right.

Second, some students reported that in the meantime they gained further surgical experience during their training. As this is subject to individual class choices and selected career paths this is not distributed equally over the cohort and represents an uncontrollable bias for a one-year long-term repetition of our study. Therefore, long term skill perception can only be obtained via self-reporting. However, there is a robust reporting of high Likert-scale values for skill retention.

## Discussion

‘Simulation based medical education (SBME) is a highly desired component of Emergency Medicine (EM) residency training programs as it allows learners to develop necessary knowledge, skills, and attitudes without exposing patients to unnecessary risk’ [18]. Ziv et al. even see simulation-based medical education as an ethical imperative [16].

### Critical evaluation

Our approach has proven to enhance the medical students’ abilities to act safely and confidently in the OR. The differentiation in training focus – emergency communication vs. hemostasis – revealed distinct benefits, with the former improving confidence levels and the latter enhancing performance metrics. A balanced curriculum incorporating both aspects could maximize students’ preparedness for OR challenges and patient safety. In combination with hands-on training, the students could consolidate their knowledge in the retest-scenarios, taking advantage of the opportunity to avoid previous mistakes. However, the pretest-retest scenario itself is a confounder, as a repetition of any task might lead to better results irrespective the intervention. We did not quantify this effect.

#### Realism of the scenario

The realism of the scenario departs from clinical accuracy. This is a shortcoming of our simulation, but it suits the simulation’s educational objectives. It serves to underscore the critical skills of decision-making in the operating room (OR) with limited information.

#### Fidelity of the phantom

The design of the phantom intentionally lacks anatomical fidelity. This decision was made to focus on fundamental surgical skills rather than on a specific organ. The simulation focusses on generic surgical techniques such as suturing, dissecting, and knot-tying in an arbitrary body cavity. This abstraction ensures that prone and averse students can benefit equally.

### Study design

The group sizes were adequate for our study objectives. However, implementing prospective randomization and propensity score matching would have been ideal to further enhance the rigor of subject allocation. Randomization was not possible ex ante; however, structural equality of the reciprocal control groups was proven ex post.

### Remark on confidence and competence

Confidence levels were visually tested against every surgical performance metric. There was no perceivable correlation. Thus, we cannot assume that a confident participant excelled the test, neither could we predict that a low-level confidence student would fail. In two metrics, there is a slight trend: Members of the communication subgroup reported higher confidence levels, the less time they took to stop the surgical bleeding (y = −0,7498× + 4,639), and members of the bleeding subgroup reported higher confidence levels, the less time they took to stop the diffuse bleeding (y = −0,671× + 4,8944). However, it is inadequate to derive causes and effect. The students might be quicker in bleeding control, because they are more confident, but they might be more confident, because they (luckily?) stopped the bleeding faster. Finally, our study cannot derive a correlation between confidence and competence. However, we feel that students feel more confident about their action when they are good at it.

### Comparison to the current state of knowledge

Our curriculum’s emphasis on simulation and online learning mirrors the findings of Gable et al., who observed confidence improvements among medical students through ‘Disaster Day Simulation Training.’ [10]. This comparison highlights the utility of simulations in resource-constrained environments and aligns with the principle of utilizing the WHO Checklist to ensure all necessary resources are available, enhancing patient safety and surgical efficacy [15]. Although our phantom operation led to an emergency, all the necessary means to control the situation were available to the students. Simulation training in a very special environment was tested on students by Padaki et al. Their ‘In-Flight Medical Emergencies’ compared the pre-test-retest scores of medical students being confronted with simulated medical emergencies on an airplane [11] with good reception of the participants. Simulation-based training has proven highly effective for improving performance and confidence in various surgical contexts. Feins et al. (2016) demonstrated that deliberate practice in a structured simulation environment significantly enhanced surgical proficiency and patient safety during cardiac surgery [19]. While our curriculum involved fewer repetitions and less depth of practice, the principles of simulation for skill enhancement are consistent. ‘Operation Bushmaster’ was a leadership curriculum for medical students. The participants performed on a 5-day field practicum of war-like situations. Of the 10 leadership lessons learned, adaptability, initiative, communication, competence, learning from mistakes, and followership are needed to complete our phantom operation successfully [12]. Fortunately, these play well with the competencies demanded by the National Competence based Catalogue of Learning Objectives [14]. It demands of the students to fulfill professional roles like the surgeon as a team member, as a professional able to perform practical tasks, being knowledgeable in therapeutic principles and emergencies. Even a short training in checklists can improve patient safety [20].

Franc-Law et al. [21] demonstrated that simulation in a disaster setting improved decision-making speed and command-and-control skills, which aligns with our goals of preparing students to handle emergencies in the operating room. Their study shows a clear link between structured simulation and performance improvement under pressure [21].

Facing our phantom operation the first time, the participants were unconsciously incompetent [1]. They were confronted with their shortcomings not knowing the skills they were lacking. After the debriefing of the first phantom operation, they became consciously incompetent. Thereafter, they worked through the digital online content of our curriculum becoming consciously competent. However, unlike in the simulation study ‘CABG with EASE’ from Korte et al. [6], we cannot claim to create experts in these skills, as the number of repetitions and the depth of deliberate practice were too shallow. Therefore, the learning level ‘unconsciously competent’ could not be reached by our curriculum.

Burkhart et al. [22] found that cardiopulmonary bypass simulation significantly improved thoracic surgery residents’ confidence and skills in managing complex perfusion scenarios, reinforcing the importance of scenario-based practice in building essential competencies [22].

Jebran et al. [23] similarly demonstrated that a physical reality simulator for minimally invasive mitral valve surgery (MIMVS) provided significant long-term skill retention, showing that repeated simulation sessions are crucial for mastering complex techniques [23].

Jasper et al. [24] emphasized that disaster preparedness training for medical students is essential for bridging gaps in clinical practice. Their study showed that simulation-based curricula significantly improved the skills and confidence of medical students, demonstrating the importance of such approaches in preparing future physicians for emergencies, including surgical settings [24].

Nes et al. built confidence in 3rd year medical students focusing on communication and conflict management awareness [25]. OR team members need to know how to speak up for the patient and when, irrespective of assumed hierarchy. When no obvious reason is present for the deterioration of a simulated patient’s condition, this is perceived as a thread and leads to insecurity. This ambiguity shall present an opportunity to learn; a challenge, rather than a threat [26]. We aimed at the students’ becoming aware of their insecurities. Hands-on teaching is seen superior to e-learning, especially in training for emergencies, as Everson et al. report in a study of recognizing a patient with simulated anaphylaxis [13].

Finally, Shipper et al. complained about the low application rates to general surgery [27]. They identified ‘comfort in the OR’ and ‘operative experience’ as important topics for applicants. We seemed to address general needs in medical students and contribute to a good perception of surgery in medical training. In conclusion, our curriculum reinforces the critical role of simulation and online education in enhancing the confidence and competencies of medical students, promising to elevate the standards of surgical training.

## Implication

By implementing a pre-test-retest phantom operation, similar training methodologies could enhance the preparedness and response efficiency of resident surgeons when faced with real-world emergencies. The findings advocate for the broader application of simulation-based training programs across various levels of medical education, emphasizing the importance of continuous learning and skill enhancement even after entering professional practice. Meanwhile we train our residents likewise.

## Supplementary Material

Appendix.docx
