# Supplementary material for: Surgical simulation in emergency management and communication improves performance, confidence, and patient safety in medical students
Source: Med Educ Online. 2025 Apr 8;30(1):2486976. doi: 10.1080/10872981.2025.2486976 (PMC11983577; doi:10.1080/10872981.2025.2486976)
Supplement: Appendix.docx [file ZMEO_A_2486976_SM8942.docx]

Appendix

# Videos

1. Preoperative preparations and WHO Checklist

<https://www.youtube.com/watch?v=uMbhxw6rMo8>

1. Instruments

<https://www.youtube.com/watch?v=9f_2V18ubQw>

1. Assisting in Surgery

<https://www.youtube.com/watch?v=6fhjHnbWQBM>

1. Stop the Bleeding

<https://www.youtube.com/watch?v=xUdv53YE6Q8>

1. Emergency Communication

<https://www.youtube.com/watch?v=bPSwfC7exbE>

1. Postoperative Care

<https://www.youtube.com/watch?v=OI1TV6P-1dc>

Surgical Knot

<https://www.youtube.com/watch?v=O866i17fS4E>

# Figure Captions as a list

Figure 1: surgical phantom trainer: 1. Knotting Task, 2. Preparation Task, 3. Sewing Task, 4. Hose for Diffuse Bleeding, 5. Vent for Hose for Surgical Bleeding, 6. Camera.

Figure 2: Phantom Operating Room Setting. Multi angle supervision allowed insight and feedback.

Figure 3: Study course. Students were assigned to a communication group or bleeding group. Their study course was identical, despite a special training in emergency communication or bleeding control, respectively.

Figure 4: Sequence of the Simulation: Tasks and bleedings. „Cut“ and „end of surgery“ were carried out on the surgeon’s command. The first successful knot during task 1 was the trigger for the surgical bleeding. The first cut during task 2 was the trigger for the diffuse bleeding. Every possible supportive measure (green boxes) was available at any time. Effectiveness is labeled as: adequate √, supportive but not adequate (√), unsuccessful x. Simulation was failed if the bleeding source remained undetected and/or no solution was applied.

Figure 5: Boxplots of clustered confidence (left), blood loss (middle), and skin to skin time (right). Legend: striped = pretest, white = retest results.

Figure 6: Change in confidence from pre-test to retest. The number of participants is illustrated in each pre-test retest group. Vectors show change of confidence. When there was no change in confidence, no vector was drawn.

Figure 7: Boxplot of the online test results. White = preoperative care, 11 points max; dotted = surgical instruments, 13 points max, vertical lines = assisting in surgery, 5 points max; horizontal lines = bleeding management and communication, 15 points max; diagonal lines = postoperative care, 3 points max. Left boxes show pretest scores, right boxes show posttest scores. In between tests, the participants watched a video of the respective topic.

# Figures

*
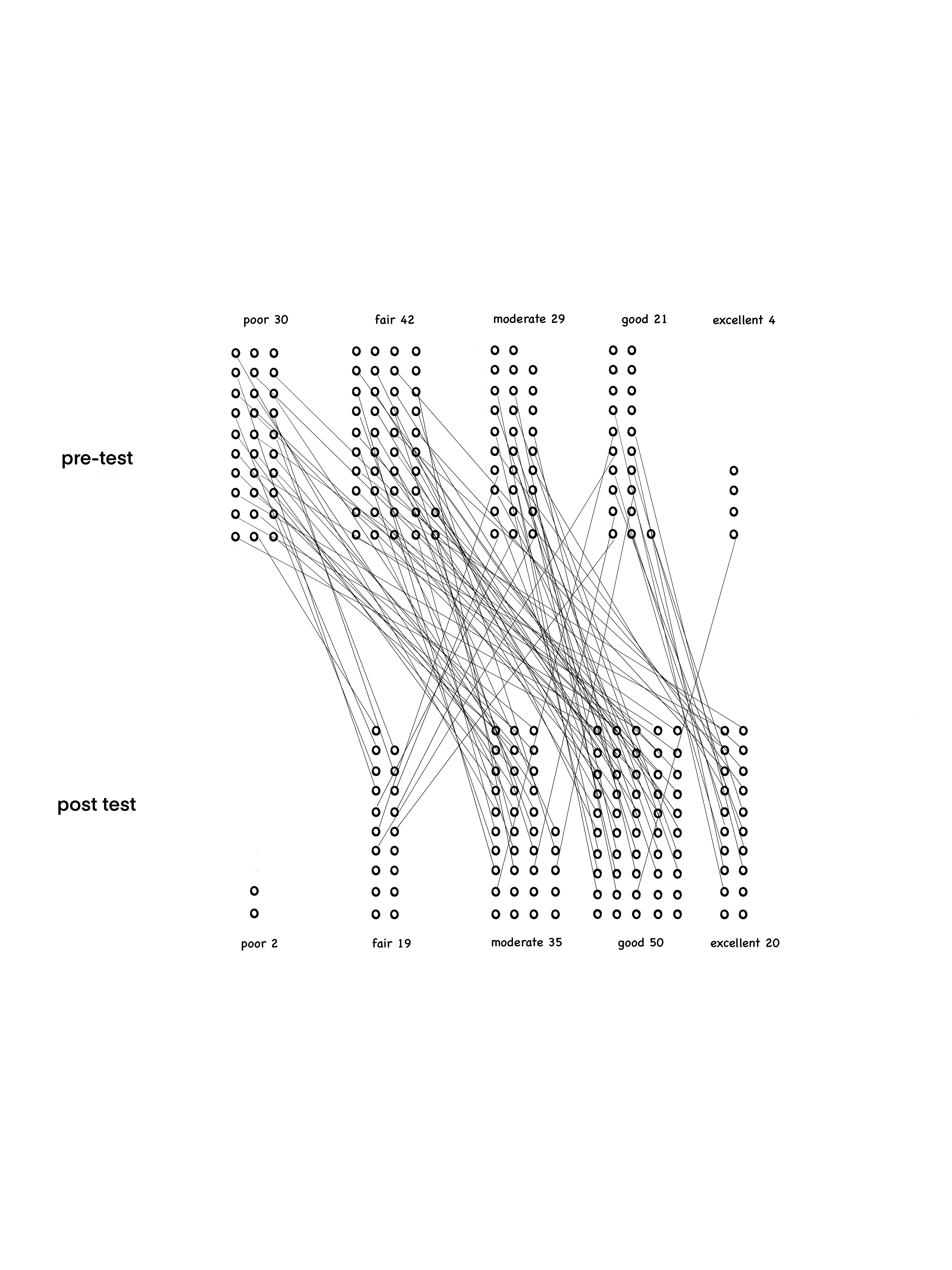
*

Figure 6: Change in confidence from pre-test to retest. The number of participants is illustrated in each pre-test retest group. Vectors show change of confidence. When there was no change in confidence, no vector was drawn.


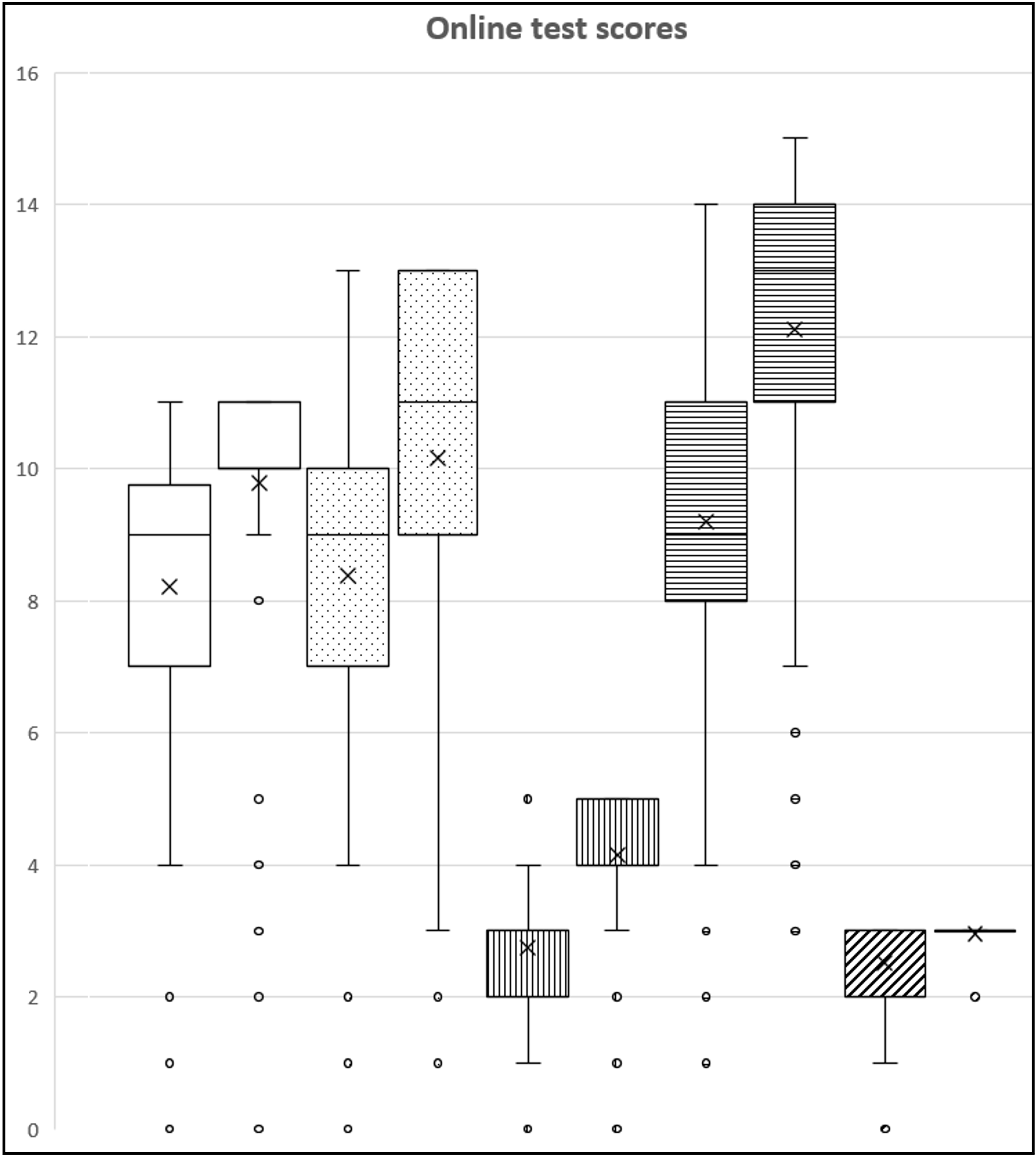


Figure 7: Boxplot of the online test results. White = preoperative care, 11 points max; dotted = surgical instruments, 13 points max, vertical lines = assisting in surgery, 5 points max; horizontal lines = bleeding management and communication, 15 points max; diagonal lines = postoperative care, 3 points max. Left boxes show pretest scores, right boxes show posttest scores. In between tests, the participants watched a video of the respective topic.

# Tables

Table 2: Provided surgical instruments and materials for one complete simulation run.

| Offered material | Quantity | Reusable | Disposable |
| --- | --- | --- | --- |
| Backhaus forceps | 2 | X |  |
| Péan clamp | 2 | X |  |
| Mosquito curved | 2 | X |  |
| Mosquito straight | 2 | X |  |
| Satinsky clamp | 1 | X |  |
| Needle holder long | 1 | X |  |
| Needle holder short | 1 | X |  |
| Heavy scissors | 1 | X |  |
| Tweezers long | 2 | X |  |
| Tweezers short | 2 | X |  |
| Langenbeck retractor | 1 | X |  |
| Roux retractor | 1 | X |  |
| Blunt prong wound retractor | 2 | X |  |
| Sharp prong wound retractor | 1 | X |  |
| Scalpel | 1 |  | X |
| Handheld suction bubble | 1 | X |  |
| Swabs | 5 |  | - |
| Sutures | 3 | - | X |
| Ligatures | 2 | - | X |
| Anesthesia I.V. infusion ^1^ | 500 mL | X | - |
| Volume I.V. infusion ^1^ | 500 mL | X | - |
| Blood/Erythrocyte concentrate I.V. infusion ^1^ | 500 mL | X | - |
| Clotting factors I.V. infusion ^1^ | 100 mL | X | - |

^1^ = This material was used by the anesthetist’s role. It was provided on a separate stand.

Table 3: Questions for the online videos.

| **Question / Task** | **Expected answer** |
| --- | --- |
| **1 Preoperative Tasks (max 11 points)** | |
| Name three aspect relevant for admittance to the OR. | Patient’s name  surgical site  written informed consent. |
| Name three aspects of positioning the patient on the operating table. | Expose surgical site of surgical access.  I.V. lines and drains must be accessible.  Avoid decubiti |
| Team time out. Think of five points your team needs to know just before skin incision. (The WHO checklist recommends 10) | 1. Every team member states his name and position. 2. Patient’s name and kind of surgery? 3. Patient’s allergies? 4. Expected duration of surgery? 5. Expected blood loss? 6. Availability of instruments? 7. Sterility? 8. All relevant imaging data visible? 9. Antibiotic prophylaxis given? 10. Particularities? |
| **2 Surgical Instruments (max 13 points)** | |
| Assign the correct function to each instrument.  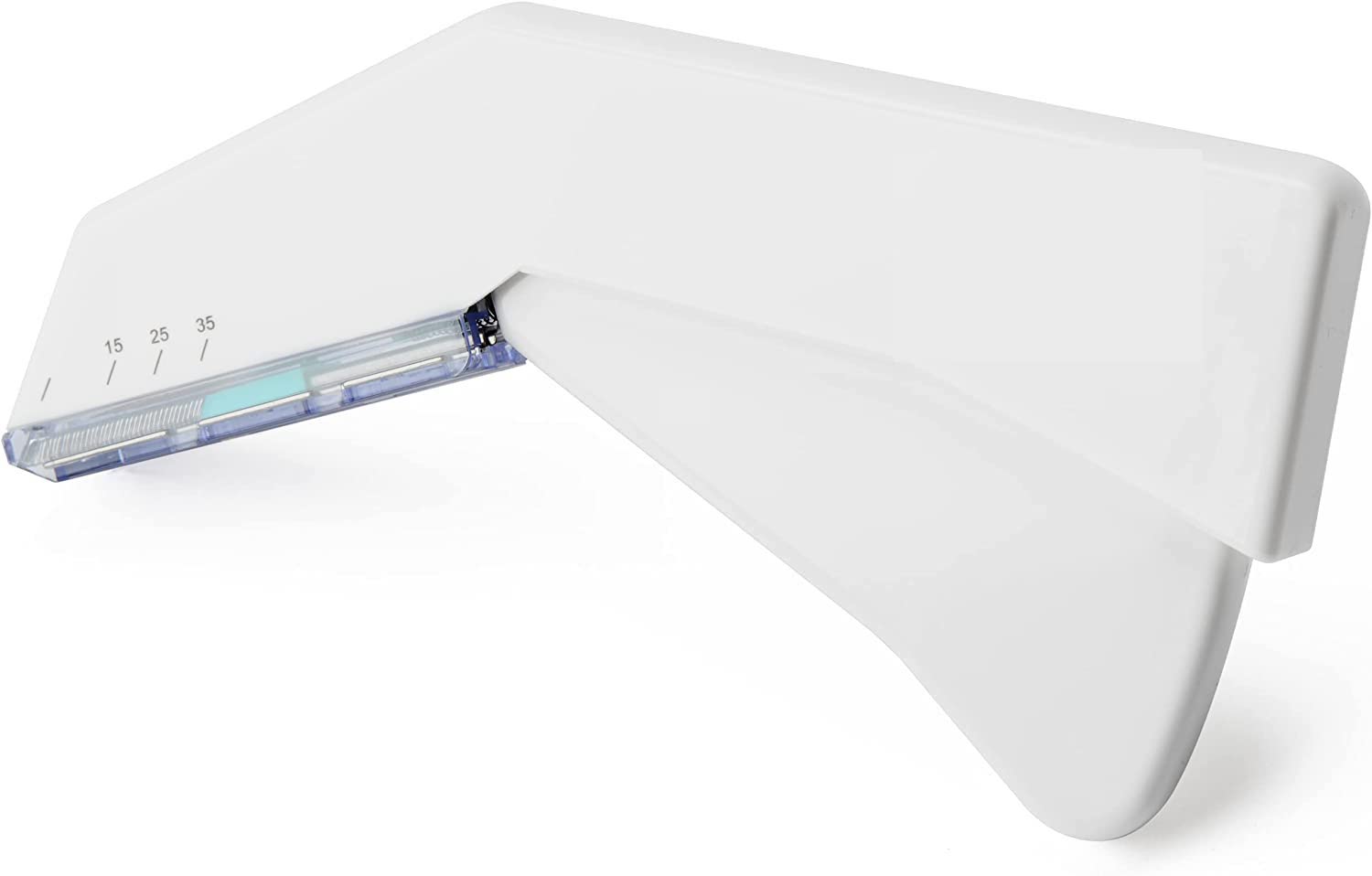  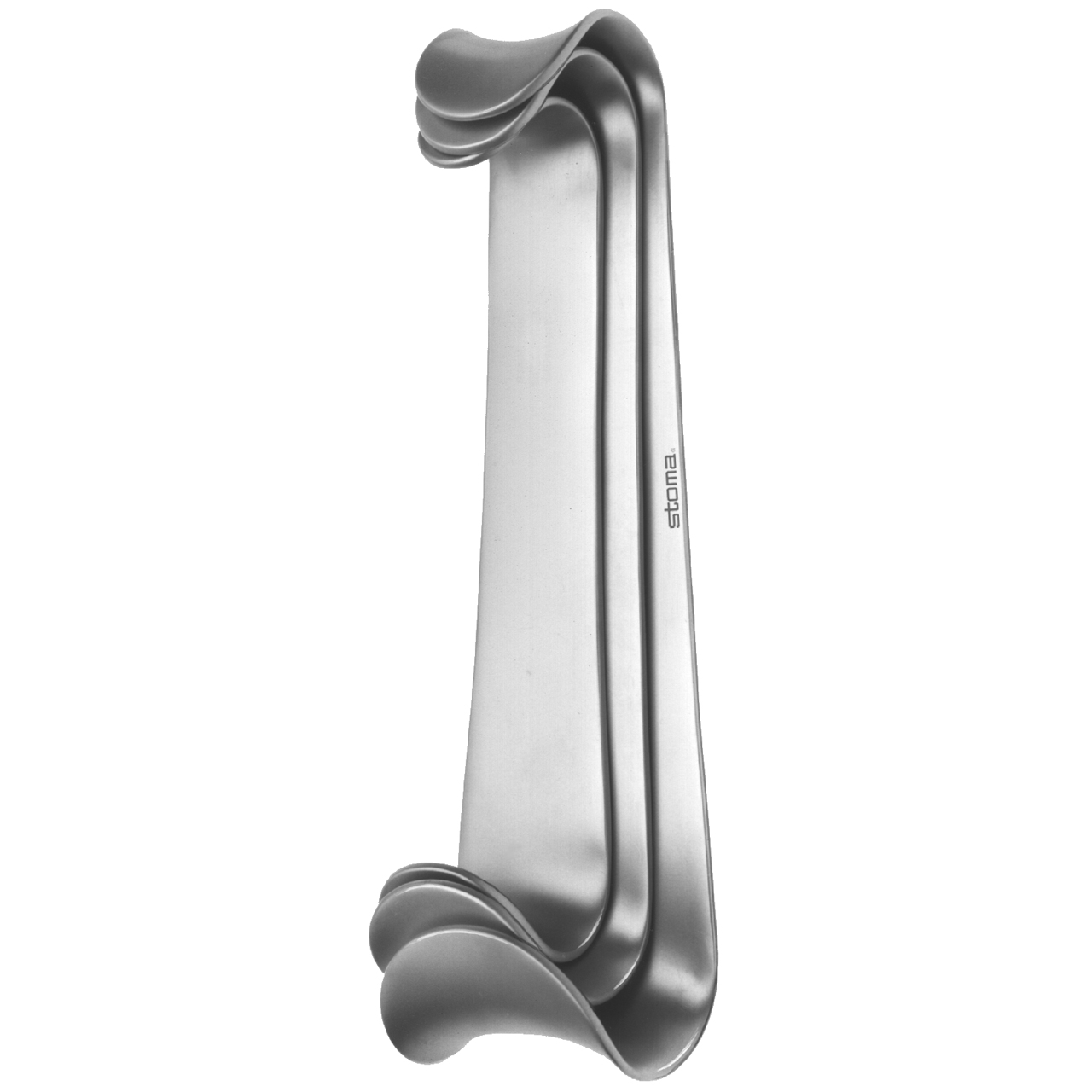  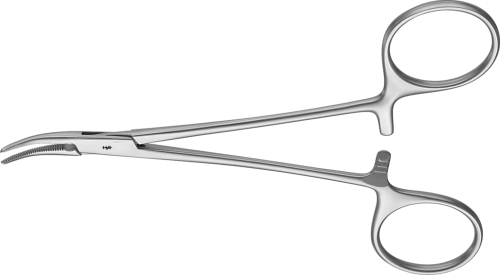  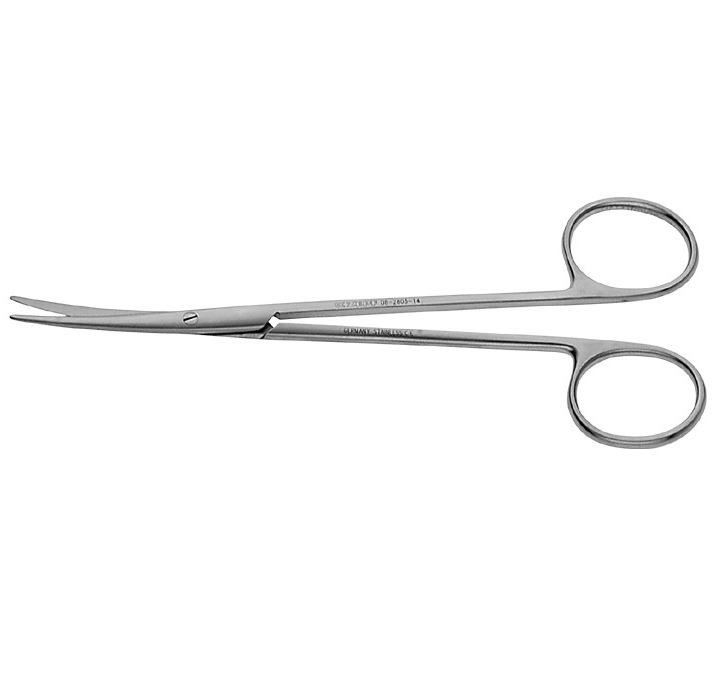 | Surgical stapling (Tissue connection)  Retraction of soft tissue  Clamping of tissue  Cutting / Dissection of tissue |
| State three benefits if the 1^st^ assistant guides the suture thread. | 1. Maintain suture tension on the tissue. 2. Avoid loops. 3. The 1^st^ assistant guides the thread in the direction of the stich, so the thread won’t saw through the tissue. |
| Assign the name to each instrument.  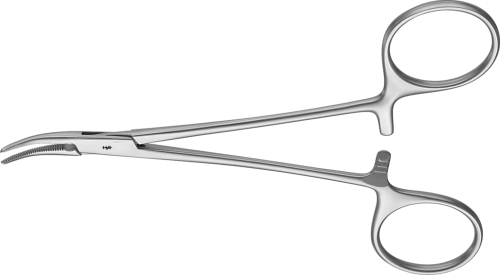  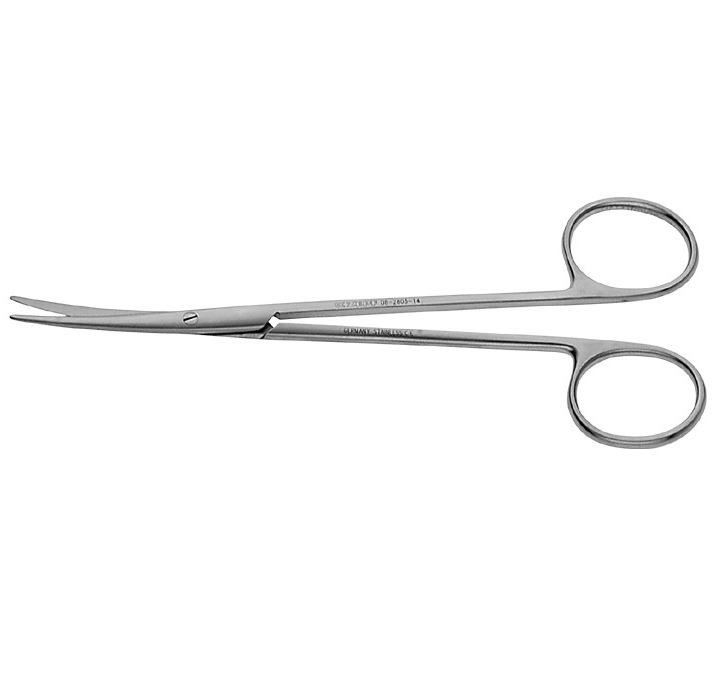  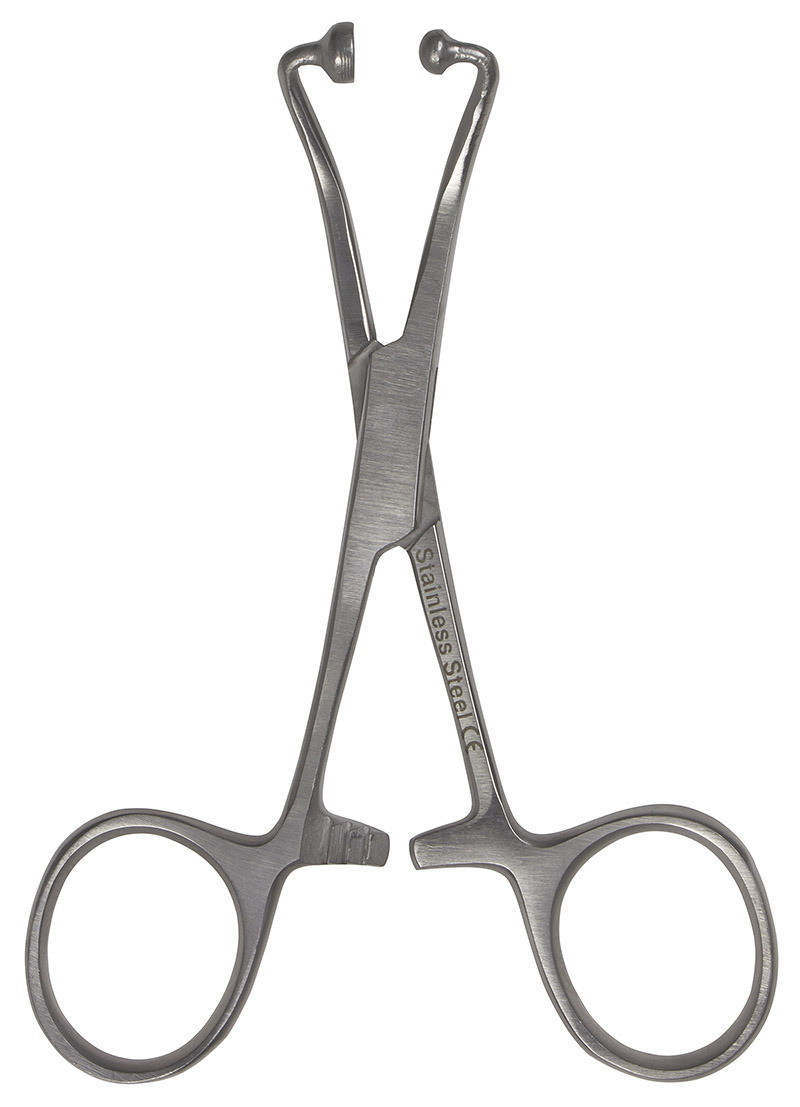  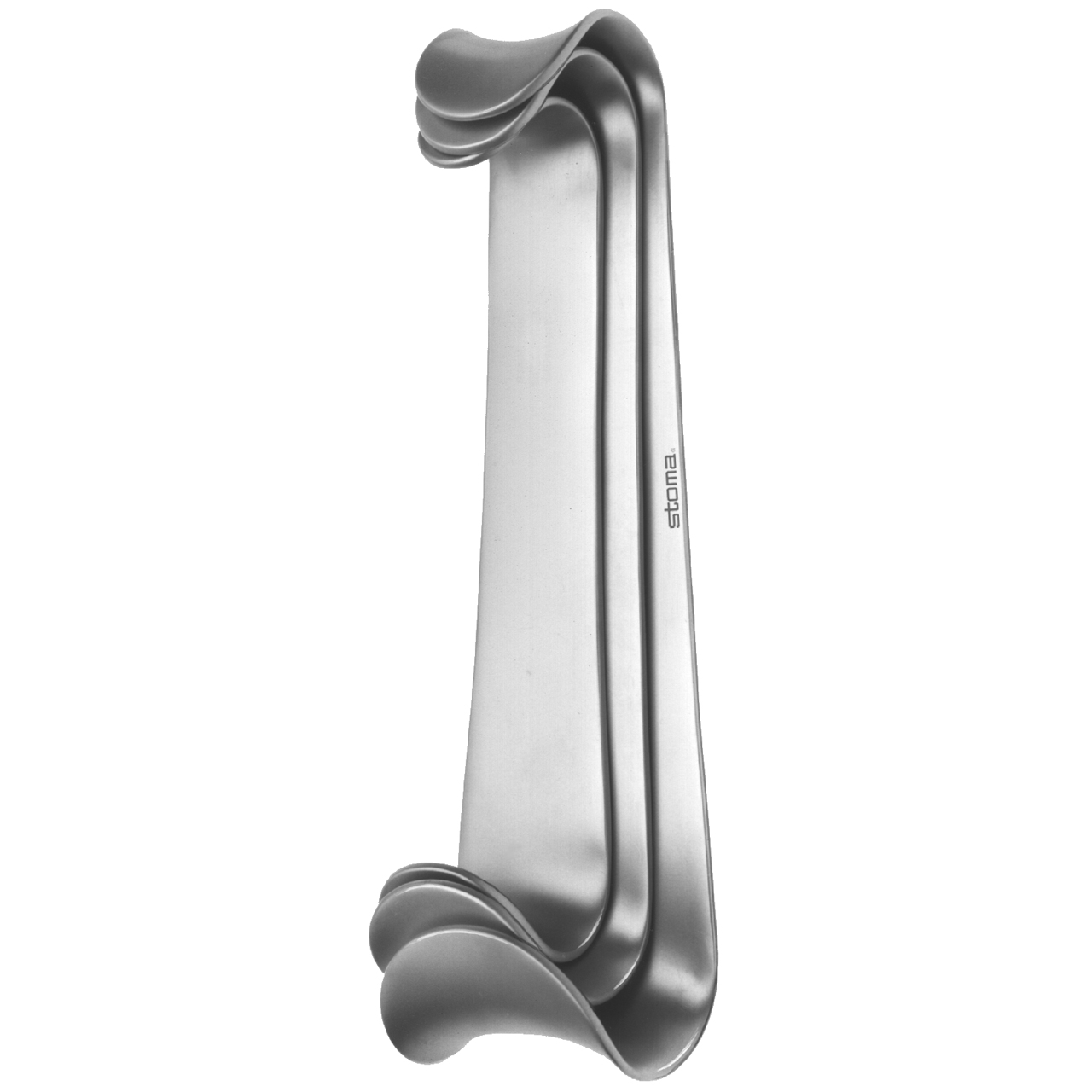  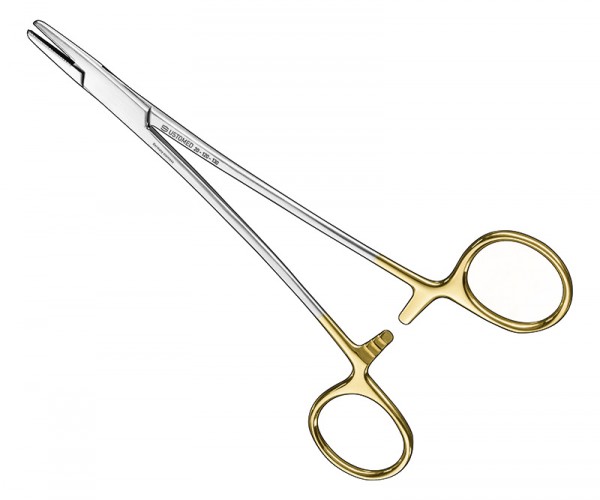  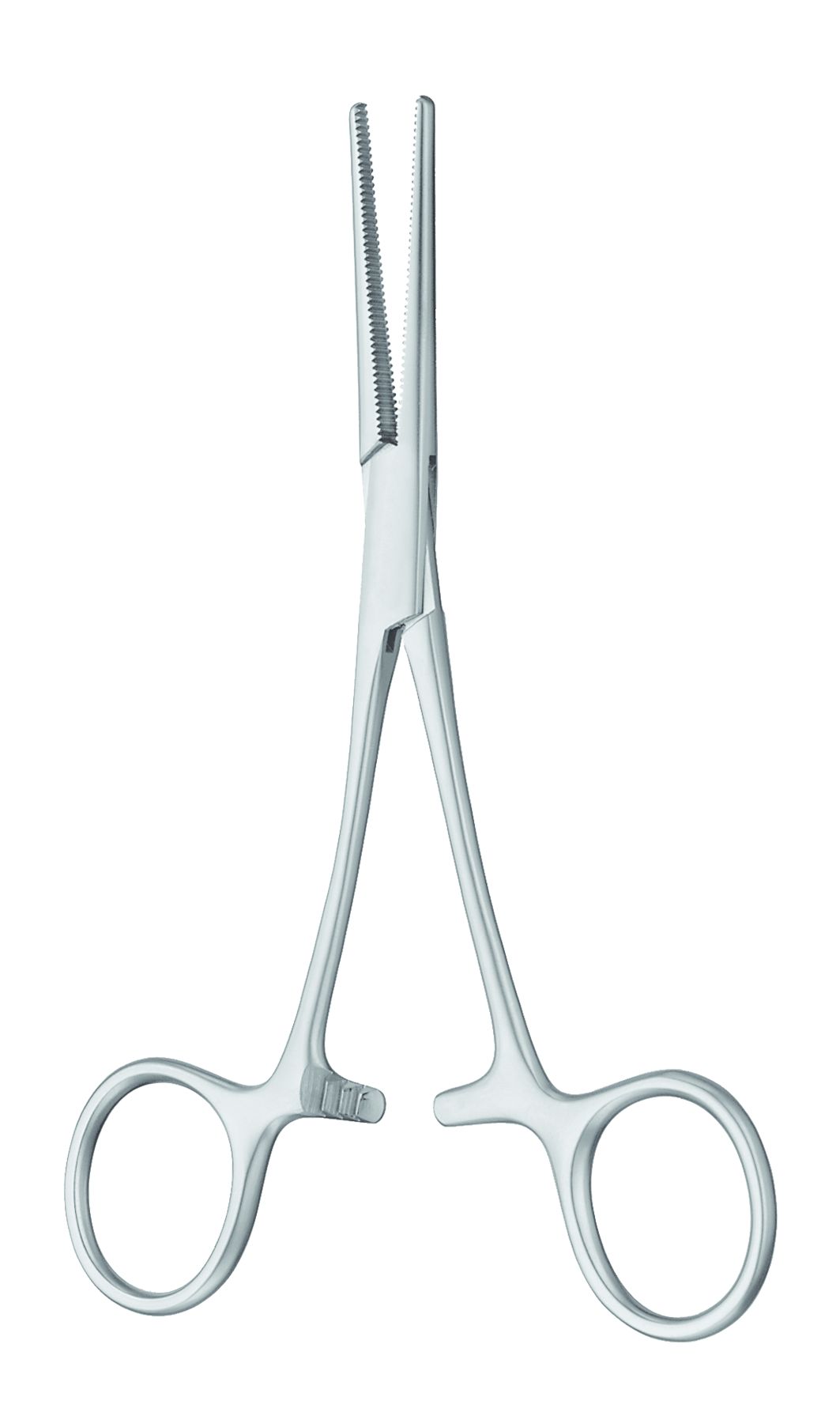 | Mosquito clamp  Metzenbaum scissors  Backhaus clamp  Roux hook  (Hegar Mayo) needle holder  Kocher Clamp |
| **3 Assisting in Surgery (max 5 points)** | |
| How would you position the patient for a left-sided resection of the upper lobe of the lung? | Lithotomy position  In a supine position  In a prone position  Struma position  On the right side (right single choice answer) |
| Place these tasks of a first assistant in the right order. (movable blocks with terms) | Check informed consent.  Positioning of patient  Surgical scrubbing  Surgical site disinfection and drapes  Team Time-Out  Assisting in surgery  Clean surgical site and dress wound.  Transfer the patient to bed |
| Name three relevant tasks for assisting in surgery. | 1. Soft tissue retraction 2. Reposition light sources. 3. Withdraw blood from the surgical site. |
| **4A Stop the bleeding (4A and B max 15 points)** | |
| Name four things you can learn from a blood gas analysis that help to estimate coagulability. | Temperature  Hb (low Hb means run out of factors)  Ca^2+^  pH |
| What situation is the safest for the patient? | 1. The surgery is led by him who feels the most in charge at the moment. 2. The surgeon will know what he is doing. 3. All team members know each other with name and function. (right answer). 4. The surgeon’s word is adamant; don’t contradict! 5. The first assistant may perform surgical tasks he has never seen before. |
| How would you treat an oozing bleeding? | 1. Cauterize, coal doesn’t bleed. 2. Always put a clamp on it. 3. Always pack with swabs. 4. Situational: pressure, cauterize, clamp, ligate. (right answer) 5. Oozing bleedings always will lessen without further ado. |
| **4B Communication in the OR** | |
| When one team member yells, everybody works worse because… | Accepted right answers.   1. Fear paralyzes. 2. Fight or flight does not solve surgical problems. 3. Muscle perfusion instead of brain perfusion. 4. Concentration gets overruled by emotion. 5. It is asymmetric communication. (Thomas Harris) |
| Think of three reasons, when a bleeding cannot be handled by the surgeon alone and anesthesia must be notified. | Accepted right answers.   1. The surgeon can only support hemostasis, but hemostasis must be sufficient. 2. Hemostasis is an enzymatic process, it needs the right pH, and temperature. 3. When a patient comes in with Hb 4 g/dl, he possesses not enough clotting factors. 4. Even if there is no bleeding at the moment, insufficient hemostasis will lead to bleeding even if the site is closed. |
| Name 3 strategies to make sure your claim was heard. | Accepted right answers.   1. Addressing the relevant team member with name or function. 2. Objectively state your message in a full sentence. 3. Value your message, “this harms the patient”. 4. Demand read back to make sure I was heard. 5. Give a hear back to show that I was heard right. |
| **5 Postsurgical care** | |
| The patient’s wound dressing is DRY but has visible signs of a past bleeding. When would you change it? | Select one right answer.   1. Every 2^nd^ day, regardless the aspect. 2. Even dry dressing needs to be changed twice/day. 3. Every 3^rd^ day, regardless the aspect. 4. No bleeding, no change of dressing. 5. At once, dried blood is not immune competent. (right answer) |
| The patient’s wound dressing is WET and has visible signs of a past bleeding. When would you change it? | Select one right answer.   1. Maybe the bleeding will stop. Then the dressing dries. 2. The patient is bleeding. He needs factor substitution at once. 3. The patient is bleeding. He needs red blood cells at once. 4. The dressing needs to be removed and the bleeding inspected. (right answer) 5. Simply change the dressing. |
| You find a wound dehiscence on the 3 POD. What do you do? | Select one right answer.   1. I assess the wound by a common scheme to find its cause. (right answer) 2. Wound swabs need 3 days. That’s irrelevant to me. 3. Maybe the wound just needs more time… 4. Even though I smell sweet sour odor, I close it with a single stich. 5. I put the patient on Vancomycin, then we can reuse the old dressing. |

Table 4: Results overview
